# A novel intelligent radiomic analysis of perfusion SPECT/CT images to optimize pulmonary embolism diagnosis in COVID-19 patients

**DOI:** 10.1186/s40658-022-00510-x

**Published:** 2022-12-05

**Authors:** Sonia Baeza, Debora Gil, Ignasi Garcia-Olivé, Maite Salcedo-Pujantell, Jordi Deportós, Carles Sanchez, Guillermo Torres, Gloria Moragas, Antoni Rosell

**Affiliations:** 1 Respiratory Medicine Department, Hospital Universitari Germans Trias I Pujol, Badalona, Barcelona Spain; 2 Germans Trias I Pujol Research Institute (IGTP), Badalona, Barcelona Spain; 3 Departament de Medicina, Universitat Autónoma de Barcelona, Barcelona, Spain; 4 Computer Vision Center and Computer Science Department, UAB, Barcelona, Spain; 5 Nuclear Medicine Department, Hospital Universitari Germans Trias I Pujol, Badalona, Barcelona Spain

**Keywords:** Pulmonary embolism, SPECT, CT, COVID-19, Radiomics, Neural networks

## Abstract

**Background:**

COVID-19 infection, especially in cases with pneumonia, is associated with a high rate of pulmonary embolism (PE). In patients with contraindications for CT pulmonary angiography (CTPA) or non-diagnostic CTPA, perfusion single-photon emission computed tomography/computed tomography (Q-SPECT/CT) is a diagnostic alternative. The goal of this study is to develop a radiomic diagnostic system to detect PE based only on the analysis of Q-SPECT/CT scans.

**Methods:**

This radiomic diagnostic system is based on a local analysis of Q-SPECT/CT volumes that includes both CT and Q-SPECT values for each volume point. We present a combined approach that uses radiomic features extracted from each scan as input into a fully connected classification neural network that optimizes a weighted cross-entropy loss trained to discriminate between three different types of image patterns (pixel sample level): healthy lungs (control group), PE and pneumonia. Four types of models using different configuration of parameters were tested.

**Results:**

The proposed radiomic diagnostic system was trained on 20 patients (4,927 sets of samples of three types of image patterns) and validated in a group of 39 patients (4,410 sets of samples of three types of image patterns). In the training group, COVID-19 infection corresponded to 45% of the cases and 51.28% in the test group. In the test group, the best model for determining different types of image patterns with PE presented a sensitivity, specificity, positive predictive value and negative predictive value of 75.1%, 98.2%, 88.9% and 95.4%, respectively. The best model for detecting pneumonia presented a sensitivity, specificity, positive predictive value and negative predictive value of 94.1%, 93.6%, 85.2% and 97.6%, respectively. The area under the curve (AUC) was 0.92 for PE and 0.91 for pneumonia. When the results obtained at the pixel sample level are aggregated into regions of interest, the sensitivity of the PE increases to 85%, and all metrics improve for pneumonia.

**Conclusion:**

This radiomic diagnostic system was able to identify the different lung imaging patterns and is a first step toward a comprehensive intelligent radiomic system to optimize the diagnosis of PE by Q-SPECT/CT.

**Highlights:**

Artificial intelligence applied to Q-SPECT/CT is a diagnostic option in patients with contraindications to CTPA or a non-diagnostic test in times of COVID-19.

## Background

Although CT pulmonary angiography (CTPA) is the most widely used imaging test for diagnosing pulmonary embolism (PE) [1, 2], another test is needed for patients with contraindications (allergy to iodinated contrast, kidney failure) or non-diagnostic on CTPA. Ventilation/perfusion single-photon emission computed tomography/computed tomography SPECT/CT (V/P-SPECT/CT) is a validated technique for PE diagnosis even in the presence of pneumonia [3, 4], but during the COVID-19 pandemic the use of ventilation has been discouraged due to the high risk of aerosol production [2, 5].

Thromboembolic disease has been a major complication in patients with COVID-19 pneumonia. In severe cases of COVID-19, an association with hyperinflammatory syndrome [6, 7] has been reported, as well as coagulation abnormalities and thrombosis [8–10]. Venous and arterial thromboembolic events occur in 31–59% of hospitalized patients with COVID-19 [8, 11], especially if they require critical care [12–16]. Diagnosis of PE in the latter situation can be challenging [17].

Due to the need to adapt the test to the safety standards required by the pandemic and in search of an alternative for patients for whom CTPA is not an option, it is worth considering perfusion single-photon emission computed tomography/computed tomography (Q-SPECT/CT) as a diagnosis alternative [18–21]. The value of Q-SPECT/CT was reported even prior to the COVID-19 pandemic [22], showing a sensitivity and specificity of 100% and 83%, respectively, for the diagnosis of PE with the Q-SPECT/CT technique [23]. Nevertheless, the diagnosis of PE can be difficult in large areas of pneumonia. In these cases, reporting takes longer and frequently needs to be validated by senior nuclear physicians to prevent errors.

The early detection of COVID-19 by medical imaging has aroused great interest within the artificial intelligence community. However, to the best of our knowledge, to date there are no published studies that use artificial intelligence to improve the diagnosis of COVID-19 PE through Q-SPECT/CT.

An artificial intelligence system to support physicians in the diagnosis of PE through Q-SPECT/CT would improve the specificity of PE diagnosis in cases of associated pneumonia and reduce the reading time of the test, and for this reason, we have decided to apply radiomics and artificial intelligence techniques as an innovative alternative that responds to the requirement of an alternative test to the CTPA to rule out PE during the COVID-19 pandemic.

Existing methods addressing the diagnosis of COVID-19 pathologies focus mainly on the detection of COVID-19 pneumonia from an analysis performed through a single modality (either X-ray or CT scans). Most methods are deep learning approaches based on well-known architectures that have proved successful in other fields (like ResNet [24], U-net [25] or EfficientNet [26], among others) that have been adapted and fine-tuned for managing COVID-19 diagnosis.

Regardless of the imaging modality and architecture, the usual approach is to use a classification scheme that provides a single diagnosis for each image/scan [27, 28]. Patients with severe COVID-19 have several lung image patterns at the same time. A main challenge is the accurate localization inside the lungs of pathologies affecting a small percentage of lung tissue (as is the case with PE).

The objective of the present work is to seek the best radiomic signature to detect image patterns of PE or pneumonia in patients with COVID-19 based only on Q-SPECT/CT. For that purpose, we present a combined approach that uses radiomic characteristics extracted from each scan and a classification network trained from scratch based on an analysis of a segmented region of interest (local analysis) of the images.

## Material and methods

### Patient recruitment

Patients were recruited from Hospital Universitari Germans Trias i Pujol (HUGTiP), a university referral hospital covering an area of 800,000 inhabitants in Barcelona (Spain).

The Q-SPECT/CT protocol is prospective and all COVID-19 patients (positive RT-PCR) were collected prospectively in 2020 (April to September 2020). In order to have a control group of patients not affected by COVID-19, we reviewed patients from a 2018 database (January–December 2018) and selected a retrospective group to ensure that they had not presented with a COVID-19 infection.

The cases were divided into two subsets, one of which was used to perform the algorithm training (training group) and the other to perform the validation (test group).

The study’s exclusion criteria were the patient's refusal to participate in the study, and the detection of severe alterations in the patients’ lungs caused by other pathologies unrelated to COVID-19 infection such as severe emphysema, bullae or interstitial lung diseases.

This study was performed in accordance with the principles of the Declaration of Helsinki. The research protocol was approved by the regional ethics committee (Ethics Committee for Clinical Research of Germans Trias i Pujol University Hospital), with the reference PI-20–161, and subjects gave their written informed consent.

### Demographics

The total number of patients included was 61, from which two cases were discarded due to technical failures in the image acquisition process or to the presence of severe abnormalities in the patients’ lungs caused by pathologies other than COVID-19.

Thus, the resulting database contained data from 59 patients, 20 in the training group (4,927 sets of samples of three types of image patterns) and 39 in the test group (4,410 sets of samples of three types of image patterns). In the training group, COVID-19 infection corresponded to 45% of the cases and to 51.28% in the test group. The demographic characteristics of each group are given in Table 1.

**Table 1:** Demographic data–patients

	Training set	Test set
Patients (*n*)	20	39
Age (average, years, SD)	60	68
Gender female, *n* (%)	12 (60%)	18 (46.15%)
COVID-19, *n* (%)	9 (45%)	20 (51.28%)
Pneumonia (*n*)	4	9
Pneumonia + PE (*n*) †	3	10
PE (*n*)	4	10
Healthy lungs	6	10
Extra categories*	3	0

^*^Extra categories: areas with abnormal CT and low perfusion; black background (areas with no tissue uptake); body tissue (areas of the body not belonging to the lungs)

^†^ All cases registered with Pneumonia + PE were cases with COVID-19

### Image data acquisition

A perfusion single-photon emission computed tomography/computed tomography (Q-SPECT/CT) based on the intravenous administration of 6 mCi (222 MBq) of 99mCt-macroaggregates of human albumin (99mCt-MAA) was acquired for each patient, with the subsequent acquisition of a tomo-scintigraphy (SPECT) and a CT in two hybrid devices indistinctly: a Symbia T2 Gamma camera (brand Siemens, based in Munich, Germany) and a Discovery NM/CT 670 ES Gamma camera (brand General Electric, based in Boston, Massachusetts, US).

The acquisition parameters were the following. The SPECT was obtained with a circular orbit with a 360º arc, 128 × 128 matrix, zoom 1, 140 keV photopeak, obtaining 90 images of eight seconds per image. The acquired CTs used 120 kV, 50–350 mA, with a slice thickness and interval of 1.25 mm (General Electric) and 3 mm (Siemens). With reconstructions of B41S, B80S, B08 and 1 soft, recon 2 lung, respectively, for CT and three-dimensional reconstruction and the format with attenuation correction for scintigraphy were used, 512 × 512 matrix.

Ventilation lung scintigraphy was not allowed due to the risk of COVID-19 cross-contamination, and ventilation alterations were therefore determined only by CT scans.

The images were extracted from PACS in DICOM format and were anonymized to guarantee the confidentiality of the data.

All the CT images obtained for the Q-SPECT/TC were reviewed by a pulmonologist, nuclear medicine physician or radiologist with more than seven-year experience, and their opinion of the expert clinicians was considered the gold standard. These experts segmented areas of healthy lung, PE or pneumonia in Q-SPECT/CT images from each patient using in-house software. For each patient scan, an expert annotated 2D regions containing pixels of one of the target tissues (healthy lungs, TEP or pneumonia). In the cases with COVID-19 pneumonia and PE, we selected and noted areas in which only one of the image patterns existed. 2D regions were identified by a single reader. The average number of 2D ROIs annotated for each patient was 25. The number of pixels of each ROI was 473.4738 on average, with a minimum of 48 and a maximum of 2,964.

**Fig. 1:**
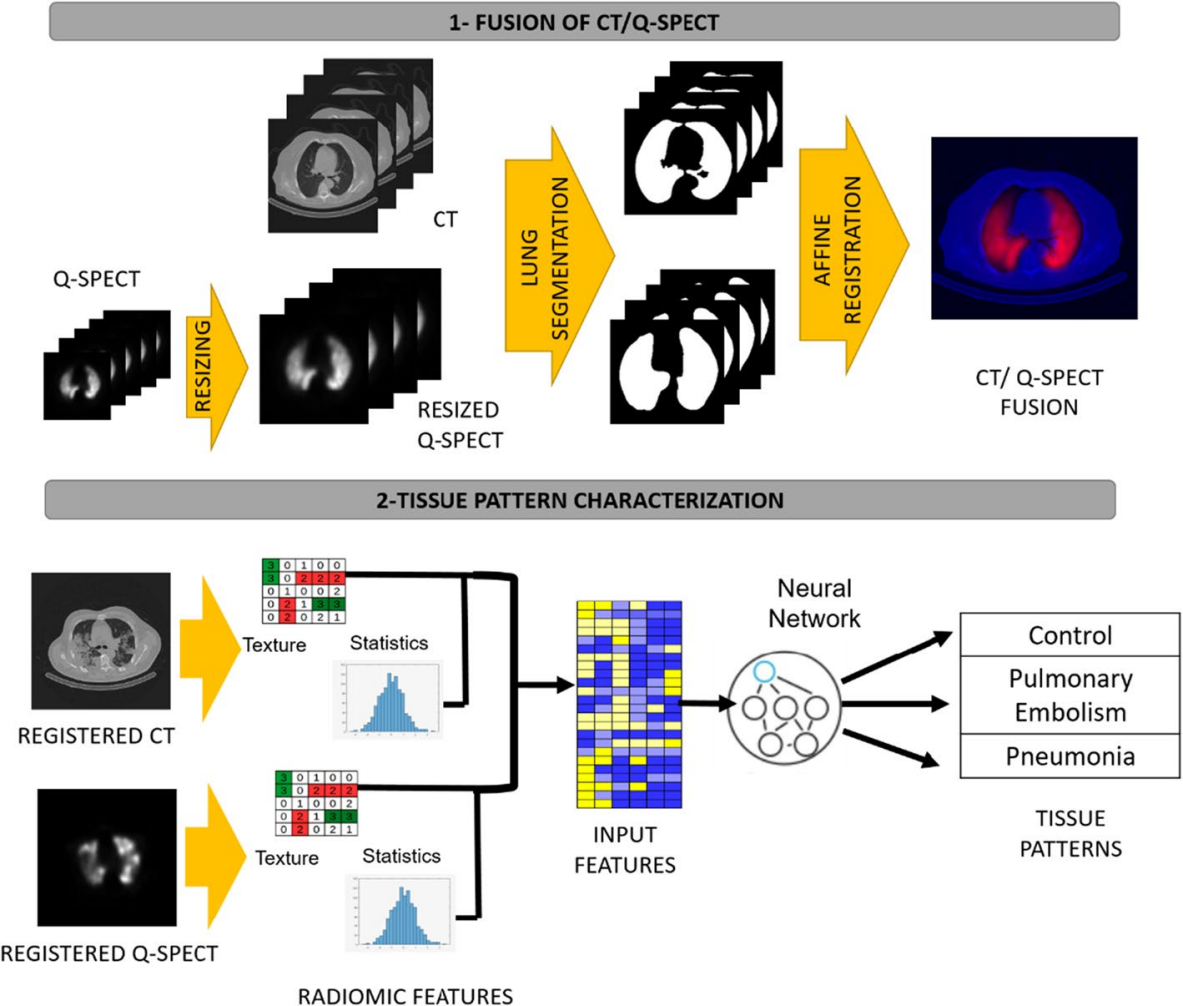
Illustrative flowchart of the stages of the system pipeline

Radiomic features were computed pixel-wise using the values of neighboring pixels contained in windows centered at each pixel in the 2D region. Each pixel in the 2D region such that a window of side equal to *sze* (in our case 3, 5 pixels) that is contained in the region is a sample to be used for either training or test. The analysis was performed upon these multiple samples and not on the final diagnosis of the patient. Since the number of samples is given by the pixels whose window is contained in the annotated ROIs, we have a different number of samples for each window size. In order to perform the statistical analysis, the data obtained for windows of size 3 × 3 pixels was resampled to match the samples obtained for the windows of size 5 × 5 pixels. The training group had 4,927 image pattern samples (2,096 healthy lung patterns, 1,204 pulmonary embolism patterns and 1,627 pneumonia patterns), while the test group had 4,410 (2.442 healthy lung patterns, 597 pulmonary embolism patterns and 1,371 pneumonia patterns) (Fig. 1).

### Image processing

Since the final diagnosis requires a combination of information from both the SPECT and CT scans, the first step consists of the registration of the two volumes in order to fuse both image modalities. In order to register volumes, first SPECT volumes were resized to match the same number of CT slices using HOROS, an open-source medical image viewer (Horos Project). Then, we used an affine unimodal [29] transformation to register a segmentation of the lungs in CT and SPECT resized volumes. By registering binary masks instead of intensity volumes, we can account for multimodal differences using the mean squared error as a cost function and thus minimize the risk of premature convergence associated with multimodal approaches using mutual information. The computed transformation was applied to intensity SPECT volumes to register them to CT scans.

The segmentation of lungs was computed using thresholding and morphological operations. CT lungs were selected as the largest connected component of the voxels with an intensity between 950 and -300 Hounsfield Units. The thresholded volumes undergo a post-processing step called morphological closing [30] that fills in interior holes of the lung mask and small concave entries at the boundary of the lung mask. The size and shape of the filled mask is given by a binary volume representing a 3D shape called a structuring element. In our case, the structuring element is a sphere of radius 5 (also refer to as size among the image processing community).

For the lung segmentation of the perfusion volumes, a threshold of intensity of 20 was selected. An intensity of 20 is over the SPECT values which reflect the amount of radioactive tracer absorbed by the lung tissue which reflects how blood is flowing within the lungs. Given that background pixels do not have a contrast agent, their baseline value is very low. Thus, voxels with an intensity over 20 were considered to be part of the lung.

The quality and interpretability of segmentations was performed visually according to the quality of the registered volumes.

In order to account for differences in SPECT intensities due to variations in contrast agent concentrations, SPECT scans were normalized using two different approaches. The normalization step was performed after the registration of volumes.

The first approach normalized SPECT values in the range [0, 1], using the maximum and minimum values of the intensities of each scan:

$$\frac{{{\text{SPECT}} - {\text{min}}\left( {{\text{SPECT}}} \right)}}{{{\text{max}}\left( {{\text{SPECT}}} \right) - {\text{min}}\left( {{\text{SPECT}}} \right)}}$$

SPECT might have spots of high concentration of the agent that are not associated with a higher lung perfusion. Those artifacts associated with the technique itself deviate maximum intensity values and introduce a drop in the intensity of normalized volumes not related to a drop in perfusion. In order to alleviate the impact of this artifact in the predictions of PE, we have also applied a normalization based on the superior quantile of SPECT intensity values:

$$\frac{{{\text{SPECT}} - {\text{min}}\left( {{\text{SPECT}}} \right)}}{{{\text{percentile}}\left( {{\text{SPECT}},99} \right) - {\text{min}}\left( {{\text{SPECT}}} \right)}}$$

For ${\text{percentile}}({\text{SPECT}},99)$, denoting the percentile leaving a right probability of 0.01 in the distribution of SPECT intensity values.

### Intelligent radiomics for the detection of pulmonary embolism

The proposed radiomic system analyzes the intensity values of CT and SPECT volumes for each voxel in order to discriminate the three clinically relevant types of image patterns defined by pulmonologist and nuclear medicine experts:

- 1. Control: healthy—normal CT and SPECT.
- 2. Pulmonary Embolism (PE): normal CT with a localized perfusion defect in the SPECT.
- 3. Pneumonia: abnormal CT with normal perfusion or with an atypical perfusion defect that does not meet PE criteria.

In order to avoid overfitting, three extra categories were added:

- 4. Areas with abnormal CT and low perfusion.
- 5. Black Background: areas with no tissue uptake.
- 6. Body Tissue: areas of the body not belonging to the lungs.

Our intelligent radiomic system was applied in three main steps. Firstly, scans were preprocessed as described in the “Image processing” section. Secondly, radiomic features selected according to their reproducibility were extracted from the registered volumes to define a radiomic feature space. Finally, a machine learning method was used to disseminate each value of the feature space between the four types of image patterns.

The radiomic features are a subset of PyRadiomics [31], an open-source python package for the extraction of Radiomic features from medical imaging volumes. PyRadiomics features include shape features, first-order features, and textural features (Gray Level Co-occurrence Matrix (GLCM), Gray Level Size Zone (GLSZM), Gray Level Run Length Matrix (GLRLM) and Gray Level Dependency Matrix (GLDM)) describing several aspects of the lesion. The subset was selected according to reproducibility against different image acquisition conditions and interobserver variability in lesion identification. Reproducibility is based on the correlation of feature values obtained from data collected using different conditions and settings [32]. The selected set of (17) features are given in Table 2.

**Table 2:** Features selected according to reproducibility

First order	Texture GLCM	Texture GLDM	Texture LGRM
Entropy TotalEnergy Uniformity	Id Idm JoinEnergy MaximumProbability	Dependence Non-Uniformity Normalized Dependence Variance Large Dependence Emphasis	Gray Level Non-Uniformity Normalized Run Length Non-Uniformity Normalized Run Percentage Short Run Emphasis

Radiomic features are computed pixel-wise using the values of pixels in the neighborhood of each pixel. These neighborhoods are squared areas (called windows) of side equal to sze (in our case 3, 5) pixels centered at each pixel of the annotated ROI. That is, for each pixel of coordinates (i0,j0) a window, w(i0,j0), of side sze is given by the pixels of coordinates (i,j) that satisfy:

w(i0,j0): = ROI(i,j), for i Î{i0-sze, i0-sze + 1, …, i0 + sze-1, i0 + sze}, j Î{j0-sze, j0-sze + 1, …, j0 + sze-1, j0 + sze}.

being ROI(i,j) the image area annotated by the expert. Given the formulation of the window used to compute radiomic features, we observe that radiomic features could only be computed for pixels such that its neighboring window pixels are also inside the ROI. We call this set of pixels samples used for both training and testing.

The radiomic features computed for each sample, using the values of the neighboring pixels contained in a square window of side sze are the input for a fully connected network which combines them in a multi-classification approach. The input for the network is the concatenation of the radiomic features extracted for the two pre-processed scans. The network has two fully connected layers with 128 neurons linked with one Relu layer and an output classification layer with sigmoid activation. To account for unbalancing in the training data, the loss function is the weighted cross-entropy given by:

$${\text{Loss}} = \mathop \sum \limits_{i} w_{i} {\text{Loss}}_{i}$$

where Loss_*i*_ is the cross-entropy loss for the i-th class and the weights, w_i_ are given by the inverse of the class frequency, w_i_, normalized to sum one:

$$w_{i} = \frac{{\rho_{i} }}{{\mathop \sum \nolimits_{j} \rho_{j} }}$$

for ${\rho }_{i}= {N\mathrm{Samp}}_{i}/N\mathrm{Samp}$ , ${N\mathrm{Samp}}_{i}$ and$N\mathrm{Samp} \mathrm{being}$, respectively, the number of samples of the i-th class and the total number of samples.

### Experiments

In order to assess the impact of the size of the window used to compute radiomic features, we trained two different model with features extracted using windows of size, sze x sze = 3 × 3 and sze x sze = 5 × 5, to compute the pyradiomics texture feature in the neighborhood of each sample. To analyze the effect of the normalization of SPECT scans, a different model was trained with data normalized using the maximum and percentile criteria for each window size. A total number of four models were trained using the configurations reported in Table 3. From now on, models will be referred to using the labels given in Table 3.

**Table 3:** Configuration of the different radiomic models

	SPECT Normalization	
Window size	Max	Percentile
3 × 3	ModelMx3: SPECT normalization based on maximum values with 3 × 3 windows	ModelPrct3: SPECT normalization based on upper percentile values with 3 × 3 windows
5 × 5	ModelMx5: SPECT normalization based on maximum values with 5 × 5 windows	ModelPrct5: SPECT normalization based on upper percentile values with 5 × 5 windows

Two different experiments were carried out:

1. *Training and Selection of Models*. A leave-1-out validation on a training set of samples from patients to select the best model for the detection of PE and pneumonia.
2. *Testing and Assessment of Models Reproducibility*. Validation of the best models on an independent set of samples from test patients to assess the reproducibility of results

### Statistical analysis

All calculations were conducted in version 15 of STATA. Sensitivity, specificity, positive predictive value (PPV), negative predictive value (NPV), area under the curve (AUC) and diagnostic accuracy were calculated using the *dt* command. The equality of ROC areas was tested using the *rocgold* command. This command independently tests the equality of the ROC area of each of several test modalities against a “gold standard” ROC curve. For each comparison, *rocgold* reports the raw and the Bonferroni-adjusted significance probability. A p-value of 0.05 was considered significant. The computation of the power and effect size of the tests was conducted using G*Power 3.1.9.2.

## Results

### Training and selection of models

Table 4 provides a statistical summary of the AUC score for the training set used for the selection of models. For each radiomic model (rows) and diagnoses (columns), we report the AUC, its 95% confidence intervals (CI) and p-values for the comparison of AUC. The *P*-value was adjusted with the Bonferroni correction. The blank cells with * correspond to the best AUC used to make the comparison.

**Table 4:** AUC statistics for the leave-1-out training for model selection

	Pulmonary Embolism			Pneumonia			Healthy Lung		
Radiomic model	AUC	95% CI	*P*-value*	AUC	95% CI	*P*-value*	AUC	95% CI	*P*-value
ModelPrct3	0.919	0.909–0.929	*	0.922	0.914–0.931	*	0.971	0.966–0.977	0.09
ModelPrct5	0.902	0.892–0.913	0.028	0.905	0.896–0.914	< 0.001	0.978	0.973–0.983	*
ModelMx3	0.835	0.822–0.848	< 0.001	0.825	0.814–0.837	< 0.001	0.928	0.920–0.937	< 0.001
ModelMx5	0.823	0.810–0.836	< 0.001	0.818	0.806–0.830	< 0.001	0.935	0.928–0.943	< 0.001

For the diagnosis of PE, models normalized using the upper percentile performed significantly better (with AUC > 0.9) than those normalized using maximum values, with ModelPrct3 performing significantly better than ModelPrct5. For the diagnosis of pneumonia, models using the upper percentile normalization also performed significantly better (with AUC > 0.9). In this case, although the analysis detected that ModelPrct3 AUC was significantly higher than ModelPrct5 AUC, the analysis of CIs suggests that this difference is not very large. In fact, according to the effect size associated with the sample size of the training set, this difference is at most 0.05. For both PE and pneumonia diagnosis, the AUC of ModelPrct3 was above 0.91, which confers percentile-based models a high diagnostic value. Regarding healthy lung, models normalized using the upper percentile performed better (AUC > 0.97), with no significant differences between them.

**Fig. 2:**
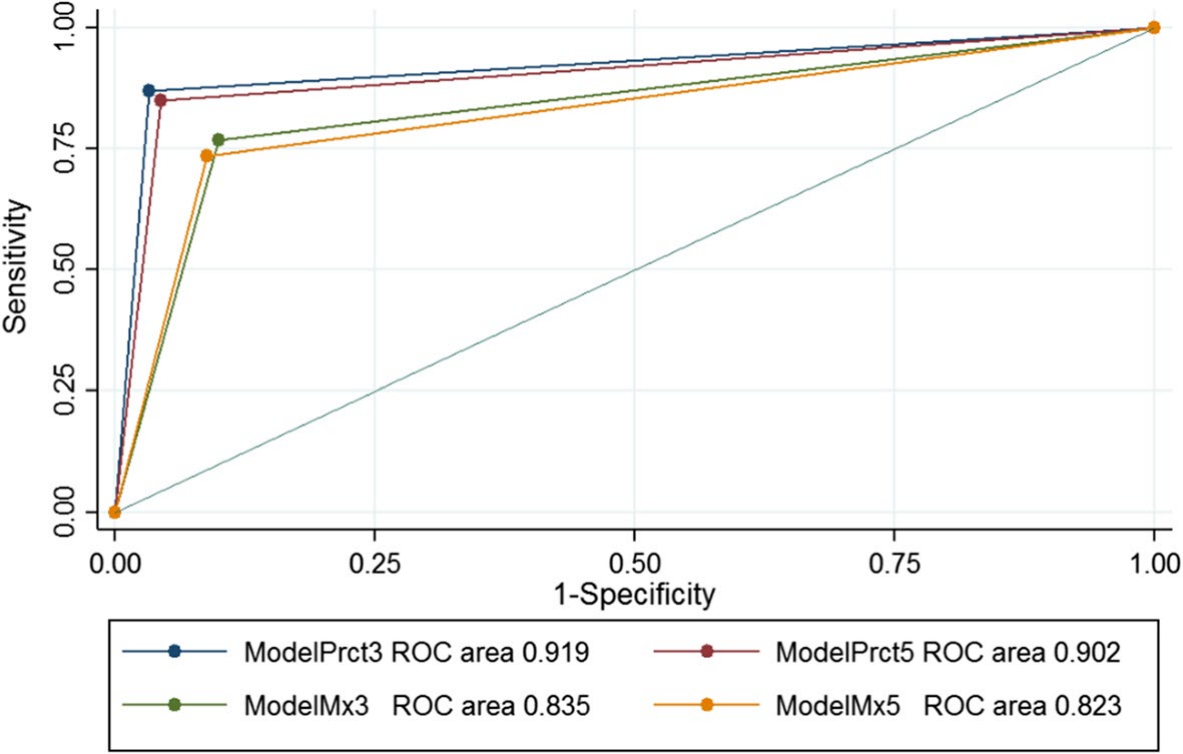
Pulmonary embolism ROC curve in the training set

**Fig. 3:**
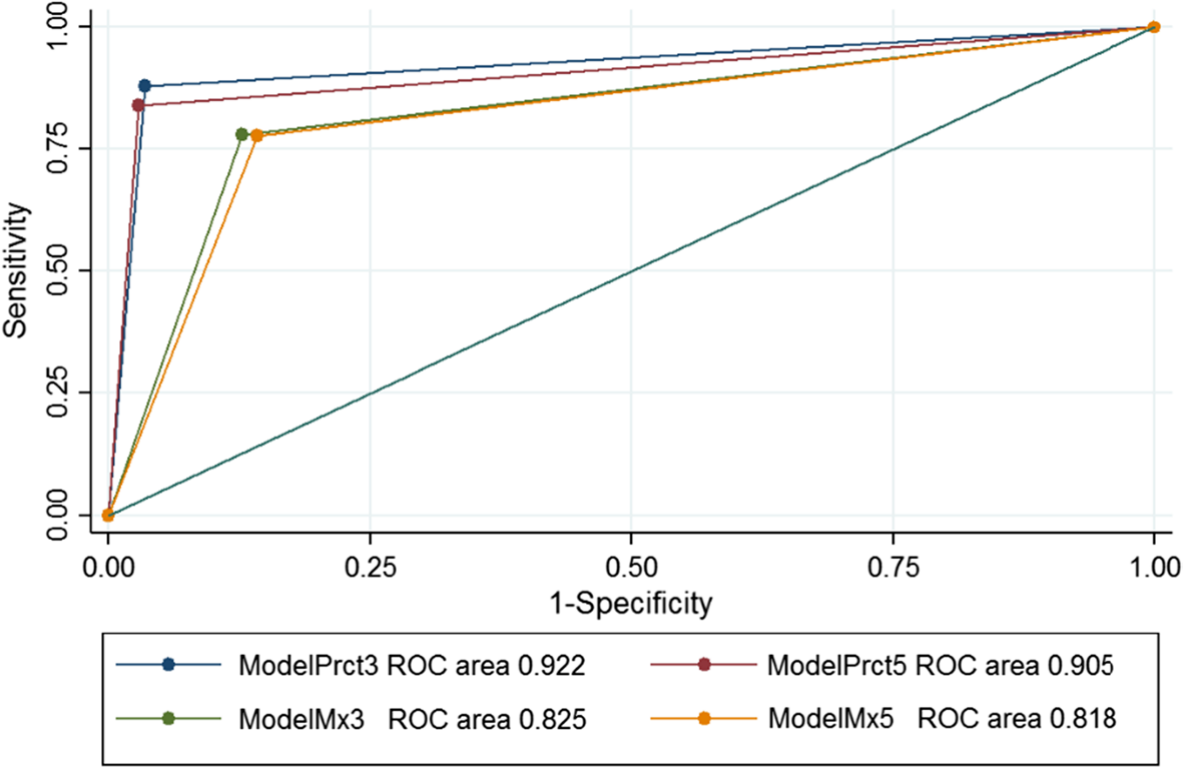
Pneumonia ROC curve in the training set

**Fig. 4:**
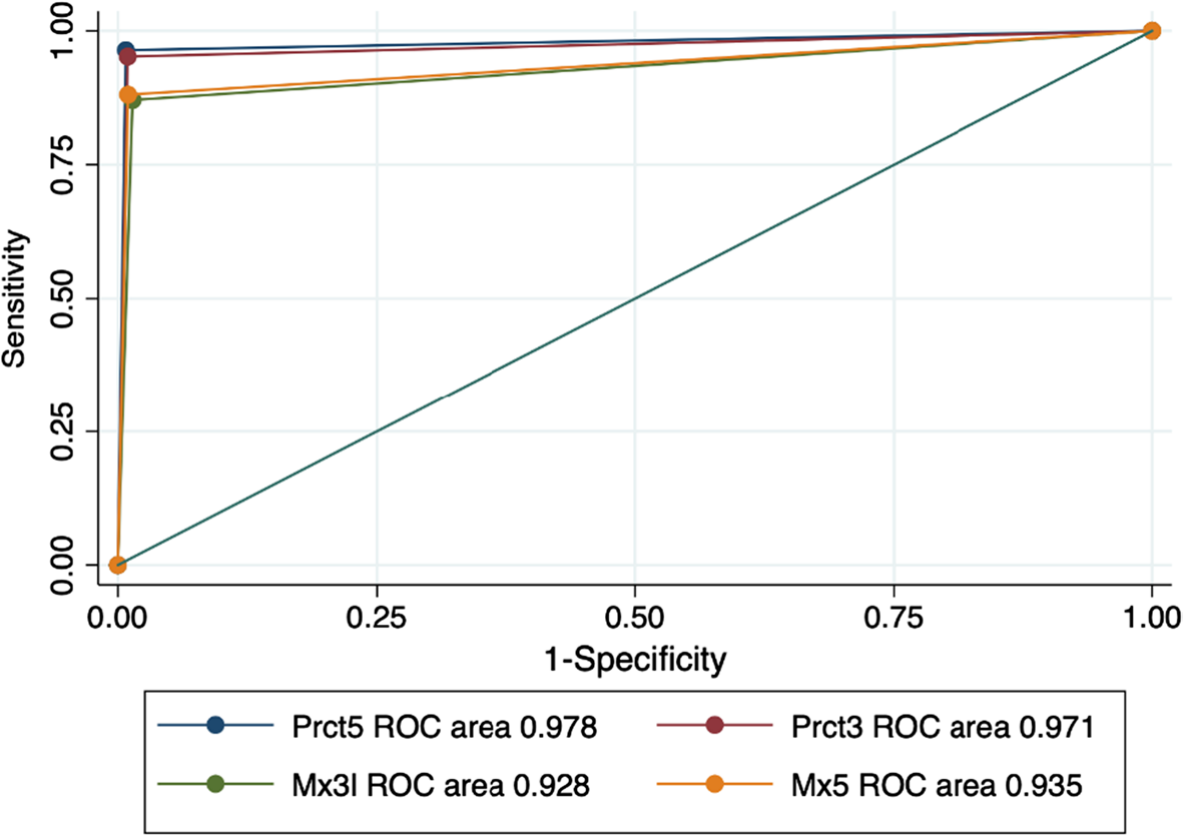
Healthy lung ROC curve in the training set

Table 5 reports the sensitivity, specificity, positive predictive value, and negative predictive value for ModelPrct3, and we also report the average values and 95% confidence intervals (CI) of each score. Sensitivity for PE, pneumonia and healthy lung were 90% or higher with a PPV of over 87%. Specificity and NPV were over 94% and 96%, respectively (Figs. 2, 3, 4).

**Table 5:** Sensitivity, specificity, PPV and NPV statistics for the leave-1-out training of the best AUC model

	Pulmonary embolism	Pneumonia	Healthy lung
Sensitivity	89.6% (95% CI 87.7–91.2)	92.5% (95% CI 91.1–93.7)	98.1% (95% CI 97.3–98.7)
Specificity	95.8% (95% CI 92.5–96.4)	94.2% (95% CI 93.4–94.9)	97.5% (95% CI 97.0–98.0)
Positive predictive value	87.0% (95% CI 85.0–88.8)	88.0% (95% CI 86.3–89.4)	95.2% (95% CI 94.1–96.1)
Negative predictive value	96.7% (95% CI 96.1–97.2)	96.5% (95% CI 95.8–97.1)	99.0% (95% CI 98.6–99.3)

### Test group

The AUC for ModelPrct3 in the test group is 0.922 (95% CI 0.090–0.935) for PE, 0.906 (95% CI 0.896–0.916) for pneumonia and 0.908 (95% CI 0.900–0.917) for healthy lung, which proves that the model predictions are reproducible.

**Fig. 5:**
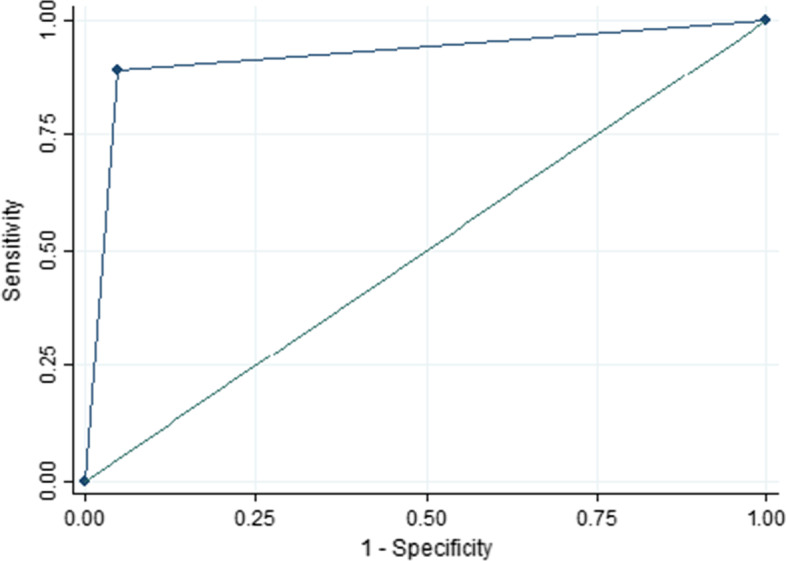
Pulmonary embolism ROC curve in the test set

**Fig. 6:**
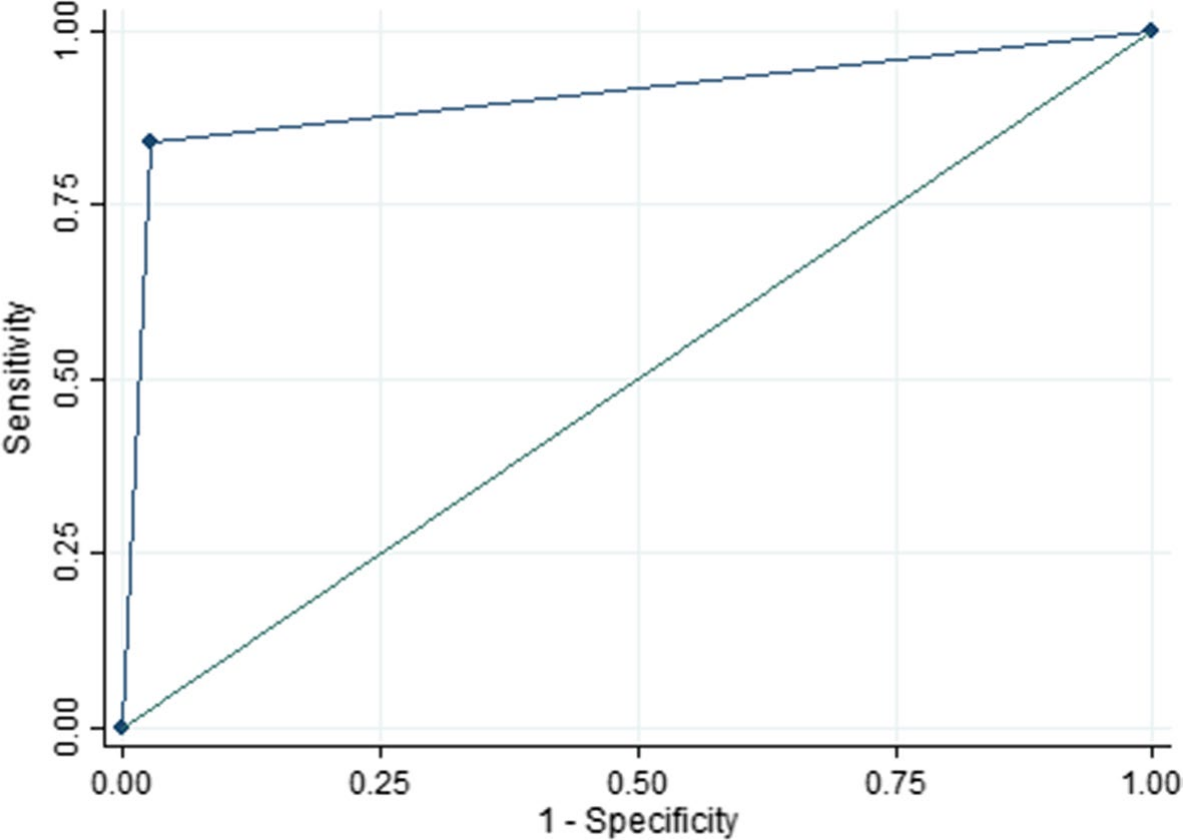
Pneumonia ROC curve in the test set

**Fig. 7:**
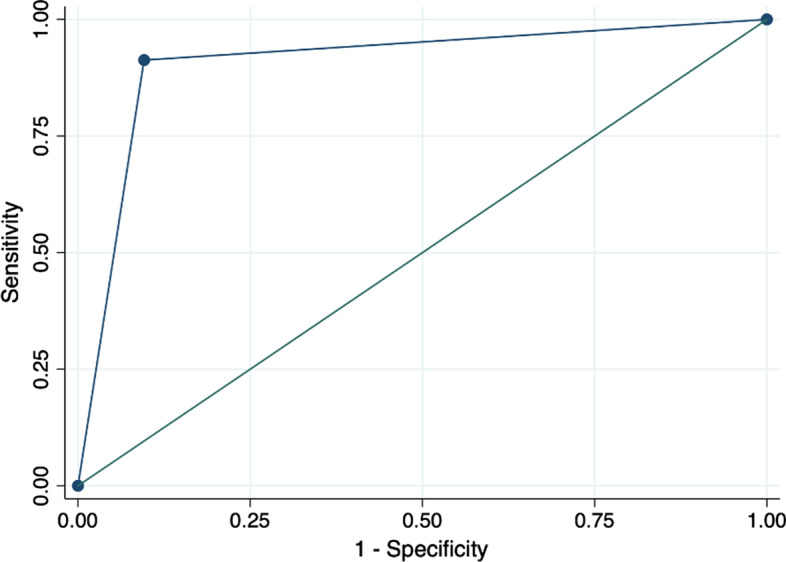
Healthy lung ROC curve in the test set

Table 6 shows the sensitivity, specificity, positive predictive value and negative predictive value for ModelPrct3, and we also report the average values and 95% confidence intervals (CI) of each score. Values for pneumonia diagnosis are almost the same as for the training set. In the case of healthy lung, although there is a decrease in the metrics in relation to training, the values are still around 90%. Values for PE diagnosis are also comparable to those of the training set, except for sensitivity, which is lower (75%), while specificity reaches 98% (Figs. 5, 6, 7).

**Table 6:** Sensitivity, specificity, PPV and NPV statistics for the test set of ModelPrct3

	Pulmonary embolism	Pneumonia	Healthy lung
Sensitivity	75.1% (95% CI 71.8–78.2)	93.3% (95% CI 91.8–94.6)	92.2% (95% CI 91.0–93.2)
Specificity	98.2% (95% CI 97.7–98.6)	93.0% (95% CI 92.1–93.9)	89.3% (95% CI 87.9–90.6)
Positive predictive value	88.9% (95% CI 86.2–91.2)	83.9% (95% CI 81.8–85.7)	91.3% (95% CI:90.1–92.3)
Negative predictive value	95.4% (95% CI 94.7–96.0)	97.3% (95% CI 92.3–93.8)	90.4% (95% CI89.0–91.6)

To further verify the diagnostic value of the proposed method, we aggregated the results obtained at pixel sample level to obtain a diagnosis for each annotated region (corresponding to a specific pulmonary lesion or region of interest). The final diagnosis is given by the most frequent image pattern on the diagnostic target: PE/noPE, Pneumonia/No Pneumonia and Healthy/not-Healthy.

Table 7 reports the sensitivity, specificity, positive predictive value, and negative predictive value for ModelPrct3, and we also report the average values and 95% confidence intervals (CI) of each score. The metrics increase for all image patterns. In particular, values for PE sensitivity increase to 85%, while keeping a high specificity very close to 90%. For the detection of healthy lung, we have a score of 100% for all metrics.

**Table 7:** Sensitivity, specificity, PPV and NPV statistics for diagnosis at lesion level

	Pulmonary embolism	Pneumonia	Healthy lung
Sensitivity	85.0% (95% CI 84.1–85.8)	95.0% (95% CI 93.9–95.6)	100% (95% CI 100–100)
Specificity	89.5% (95% CI 88.6–90.3)	100% (95% CI 99.1–100)	100% (95% CI 100–100)
Positive predictive value	89.5% (95% CI 88.6–90.3)	95.0% (95% CI 93.9–95.6)	100% (95% CI 100–100)
Negative predictive value	85.0% (95% CI 84.2–85.8)	100% (95% CI 99.1–100)	100% (95% CI 100–100)

## Discussion

The diagnosis of pulmonary embolism in COVID-19 patients is challenging. In this sense, radiomics is a tool that could help the physician in the diagnosis of PE and pneumonia, since it is able to detect different image patterns obtained by Q-SPECT/CT, without the need to perform the ventilation technique.

To the best of the authors’ knowledge, there are no published studies that apply artificial intelligence to ventilation/perfusion SPECT/CT or Q-SPECT/CT. This is an experimental study and is a first step toward a complete intelligent radiological system capable of diagnosing PE and pneumonia image patterns only by Q-SPECT/CT.

Our algorithm uses images obtained by Q-SPECT/CT and have proved to be useful to detect both PE and pneumonia image patterns. The results obtained for a training set of 20 patients using leave-1-out sampling show that the size of the window used to compute local radiomic features is not a critical parameter. In contrast, the normalization of the PE needed to compensate different concentrations of the contrast agent has a significant impact on the performance of the models, with a normalization based on percentiles being particularly recommended. The results obtained for an independent set of 39 test patients show the reproducibility of results and a high diagnosis capability and rule out for both PE and pneumonia. The sensitivity and AUC for the detection of pneumonia (93.3% recall and AUC = 0.906) is comparable to deep learning approaches using a larger number of training cases including COVNet [24] (90% recall and 0.96 AUC) and the early work of Zhang et al. [33] (88% recall and 0.92 AUC). Regarding PE, in spite of an AUC = 0.92 in tests, the sensitivity dropped to 75%. Nevertheless, with a positive predictive value of over 88%, we consider that this does not invalidate the model for PE. Moreover, the specificity and negative predictive values reach 98% and 95%, respectively, which indicates that it is an adequate technique to rule out this pathology, which is clinically useful. When the results obtained at the pixel sample level are aggregated into regions of interest, the sensitivity of the PE increases significantly to 85% but the specificity is lower (89.5%). Nevertheless, all metrics improve for both pneumonia and healthy lung. These results suggest that aggregating the results into regions of interest provides the clinician with a better tool for diagnosing PE.

In relation to the working approach, an artificial intelligence diagnosis based on the analysis of whole images/scans might only detect the main pathology while ignoring secondary ones which are also clinically relevant. For this reason, the local analysis approach adopted by this study is important. Another issue is the clinical interpretability of results. Although interpretability can be improved with the use of a heatmap (such as gradient-weighted class activation mapping [34]), deep learning approaches are still difficult to interpret and lack the ability to accurately locate the injured tissue. Another recently identified concern [35] is the sensitivity of models to the quality and quantity of the cases used for training and testing, which can lead to overestimating the results. Deep learning methods require a large number of annotated images for training. This is not a major issue in most fields of application, but, unfortunately, there is a limited availability of images in the case of COVID-19 patients. Moreover, it is suspected that its protocol of acquisition may introduce bias in models [36]. In particular, it has been reported [37] that the high-performance of machine learning methods could be attributed mainly to the presence of image patterns (such as corner labels or instrumentation), device acquisition parameters or population factors (such as sex or age). If these characteristics are specific to some of the classes (groups of patients), models may learn to recognize these biases in the data set, rather than focusing on the pathologies they are trying to detect. This bias, of course, limits the generalization and reproducibility of results when tested on data sets with a different origin from the ones used in training and testing. We believe that adopting a local approach that analyzes small regions rather than the entire scan could minimize the need for a large number of annotated cases, as well as reducing the impact of image bias.

This study has some limitations. First, the local identification of image patterns must be added for each case (similar to [24]) to produce a multiple clinical diagnosis and to be able to produce a global diagnosis. Second, although the training group and the test groups are different, the models must be tested in cases originating from other hospitals to fully validate the generalizability and clinical applicability of the models. Third, we have excluded cases with emphysema and pulmonary fibrosis due to the difficulties involved in interpreting Q-SPECT/CT without ventilation, and for future studies we will include this type of cases in order to be able to make a useful algorithm in real life. Fourth, despite the fact that there are many samples analyzed, the number of patients is small, so for future studies we must have a larger number of patients that allows us to consolidate our algorithm. Finally, a model based on 2D radiomics, though similar to a histological analysis of the lesion, might not reflect 3D aspects of the lesion, like the spatial distribution of tissue patterns and volumetric measures of the extent of each pathology. However, given that the goal is to detect the presence of either TEP or pneumonia, this is not a main limitation for this particular study. For the computation of the extension and volume of the diseased lung tissue a 3D analysis should be carried out. This could be done by either accumulating the presented 2D radiomics or training a 3D radiomic model using 3D ROIs.

The main strength of this study is the novelty of using artificial intelligence to focus on PE and COVID-19 pneumonia diagnosed with Q-SPECT/CT images, which may provide a diagnostic alternative for patients for whom CT pulmonary angiography (CTPA) is not capable of diagnosing PE or for when the technique is contraindicated. It is important to mention that it also provides a safer alternative to V-Q/SPECT-CT by avoiding aerosol contamination that occurs in ventilation, preventing the spread of diseases such as COVID-19. Although our system does not provide a global diagnosis of the patient, it is capable of classifying different image patterns and identifying areas affected by PE, as well as providing reliable information on the areas affected by pneumonia and healthy lung areas.

Our next step is to analyze the impact of our method on the final diagnosis of patients and to apply and adjust this algorithm to the current practice of V/Q-SPECT for PE not associated with COVID-19 and to include all types of respiratory diseases (emphysema, pulmonary fibrosis).

Our ultimate goal is to build a software for clinical use that can provide us with a diagnosis of PE and pneumonia only with an “intelligent Q-SPECT/CT” to be validated with our standard Ventilation/Perfusion-SPECT/CT. If this is achieved, ventilation could even be avoided, saving time and healthcare costs. This would mean a paradigm shift in the use of SPECT/CT for the diagnosis of these pathologies in times of COVID-19 or future epidemics, not only to minimize the risk of contagion by aerosols, but also to avoid unnecessary examinations and reduce test times.

## Conclusion

In summary, a combined approach based on artificial intelligence and radiomics can detect areas of pneumonia and areas of pulmonary embolism using a limited amount of annotated data. The ability to detect different image patterns in patients with COVID-19 encourages us to continue working to achieve a global diagnosis through a local analysis that allows us to develop a software for clinical use based on SPECT/CT.

## Data Availability

Anonymized data are available from the corresponding author on reasonable request.

## Abbreviations

PE: Pulmonary embolism
CTPA: CT pulmonary angiography
Q-SPECT/CT: Perfusion single-photon emission computed tomography/computed tomography
V/P-SPECT/CT: Ventilation/Perfusion single-photon emission computed tomography/computed tomography SPECT/CT
PPV: Positive predictive value
NPV: Negative predictive value
