# Correction: A novel intelligent radiomic analysis of perfusion SPECT/CT images to optimize pulmonary embolism diagnosis in COVID-19 patients

**DOI:** 10.1186/s40658-023-00532-z

**Published:** 2023-02-17

**Authors:** Sonia Baeza, Debora Gil, Ignasi Garcia-Olivé, Maite Salcedo-Pujantell, Jordi Deportós, Carles Sanchez, Guillermo Torres, Gloria Moragas, Antoni Rosell

**Affiliations:** 1 Respiratory Medicine Department, Hospital Universitari Germans Trias I Pujol, Badalona, Barcelona, Spain; 2 Germans Trias I Pujol Research Institute (IGTP), Badalona, Barcelona, Spain; 3 Departament de Medicina, Universitat Autónoma de Barcelona, Barcelona, Spain; 4 Computer Vision Center and Computer Science Department, UAB, Barcelona, Spain; 5 Nuclear Medicine Department, Hospital Universitari Germans Trias I Pujol, Badalona, Barcelona, Spain

**Correction: EJNMMI Physics (2022), 9(1):84** 10.1186/s40658-022-00510-x

Following the publication of the original article [1], the authors reported that the figures were cited and presented as part of the Background section instead of where they were originally cited in the text.

The original article [1] has been updated.
